# The Tetrapeptide HAEE Promotes Amyloid-Beta Clearance from the Brain

**DOI:** 10.3390/ijms262311591

**Published:** 2025-11-29

**Authors:** Kristina A. Mukhina, Kseniya B. Varshavskaya, Aleksandra D. Rybak, Viktor V. Grishchenko, Elena V. Kuzubova, Mikhail V. Korokin, Olga I. Kechko, Vladimir A. Mitkevich

**Affiliations:** 1 Engelhardt Institute of Molecular Biology, Russian Academy of Sciences, Vavilov Str., 32, 119991 Moscow, Russia; kristina.mukhina@gmail.com (K.A.M.); kseniya270897@gmail.com (K.B.V.); sashafish2@gmail.com (A.D.R.); viktor.grishchenko@chemistry.msu.ru (V.V.G.); olga.kechko@gmail.com (O.I.K.); 2 Research Institute of Pharmacology of Living Systems, Belgorod State National Research University, 85 Pobedy St., 308015 Belgorod, Russia; 1015artek1015@mail.com (E.V.K.); mkorokin@mail.ru (M.V.K.)

**Keywords:** Alzheimer’s disease, amyloid-beta, tetrapeptide HAEE, blood–brain barrier, microglia, Aβ clearance

## Abstract

Alzheimer’s disease is characterized by the accumulation of neurotoxic forms of amyloid-beta (Aβ) in the brain, leading to synaptic dysfunction, neuroinflammation, and neuronal death. The tetrapeptide HAEE crosses the blood–brain barrier (BBB), inhibits the formation of toxic Aβ oligomers, and reduces amyloid burden *in vivo*. However, the mechanisms of HAEE’s anti-amyloidogenic effect remained incompletely understood. In this study, we investigated the mechanism of HAEE-dependent Aβ clearance both *in vitro and in vivo*. Using ELISA, we assessed the HAEE effect on the levels of Aβ, IL-6, and TNFα in mouse brain tissue following intracerebroventricular administration. The mechanism of the anti-Aβ effect of HAEE was studied using primary brain cell cultures and a BBB transwell model through ELISA, flow cytometry, and microscopy. We showed that HAEE reduced Aβ level by 35% and IL-6 level by 40% in mouse brain tissue. HAEE enhanced Aβ clearance via LRP1- and PgP-dependent Aβ transport through the BBB and doubled the rate of Aβ degradation by microglia. In addition to inhibition of Aβ aggregation, HAEE dissolved already formed Aβ oligomers. The HAEE-induced decrease in IL-6 levels in the mouse brain was associated with reduced pro-inflammatory activation of microglia. Thus, HAEE’s effect against Aβ-related neuropathologies is realized through a decrease in the level of toxic Aβ oligomer and inhibition of neuroinflammation.

## 1. Introduction

Alzheimer’s disease (AD) remains one of the most common and socially significant neurodegenerative disorders that poses a serious challenge to healthcare systems worldwide [1]. According to the current concept, AD pathogenesis is based on a cascade of molecular events, the key elements of which include aggregation of beta-amyloid (Aβ) into neurotoxic oligomers and insoluble plaques and hyperphosphorylation of tau protein [2]. Neurotoxic Aβ species interact with numerous membrane receptors and signaling pathways that lead to synaptic dysfunction, disruption of intracellular calcium homeostasis, oxidative stress, neuroinflammation, and, ultimately, neuronal death and glial cell dysfunction [3,4,5].

Currently, the most effective therapy for AD is anti-amyloid treatment [6]. Two monoclonal antibodies targeting Aβ aggregates or its pyroglutamate-modified form are used to treat patients. Both antibodies effectively reduce cerebral amyloid load and slow the decline of patients’ cognitive functions [6,7]. However, these antibodies poorly cross the blood–brain barrier (BBB) and accumulate around cerebral vessels, which results in side effects [8]. Another drawback of antibody-based therapy is its high cost, which makes this treatment accessible only in some high-income countries [7,9]. Low-molecular-weight compounds represent a potential alternative to antibodies for anti-amyloid therapy. Mostly, such compounds cross the BBB more efficiently and have lower synthesis costs [10].

We previously showed that the tetrapeptide HAEE (Ac-HAEE-NH_2_) effectively inhibited amyloidogenesis *in vivo* [8]. Due to its low molecular weight and the distribution of its charged amino acid residues, HAEE can cross the BBB [11]. HAEE binds to Aβ at the ^11^EVHH^14^ site and blocks the formation of toxic, zinc-induced Aβ oligomers [11,12]. In addition, the tetrapeptide completely abolishes the inhibitory effect of Aβ_42_ on nicotinic α4β2 acetylcholine receptors and prevents the formation of amyloid plaques in the brains of the experimental animals modeling AD pathogenesis [13].

Thus, the efficacy of HAEE is associated, at least in part, with its ability to reduce the amyloid load in the brain. However, the mechanisms underlying this process remain unknown. In this work, we investigated the mechanism of HAEE’s anti-amyloidogenic effect *in vivo and in vitro*.

## 2. Results

### 2.1. HAEE Reduces Aβ and IL-6 Levels in the Mouse Brain and Accelerates Aβ Transport Across the BBB In Vitro

**Figure 1:**
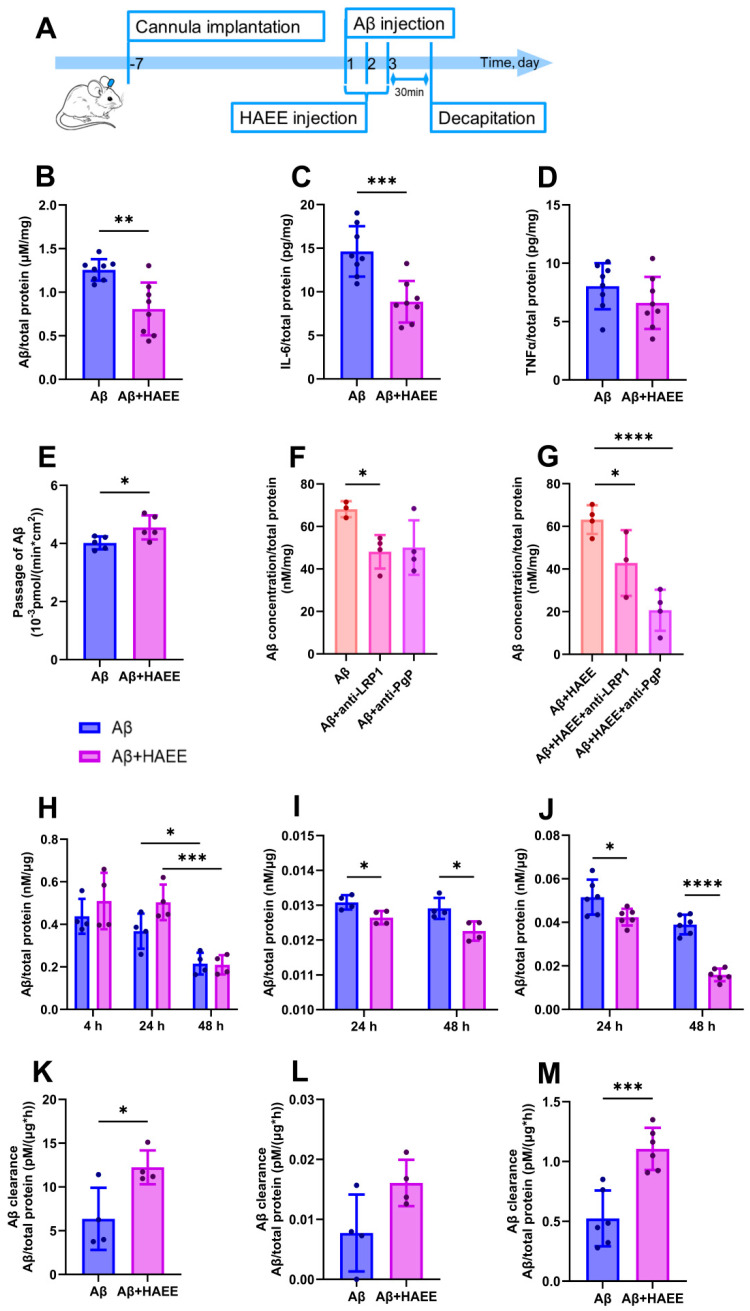
The effect of HAEE on Aβ and IL-6 levels *in vivo* and Aβ clearance in the BBB model and cell cultures. (**A**) Schematic diagram of the *in vivo* experimental design. Concentration of Aβ (**B**), pro-inflammatory cytokine IL-6 (**C**), and TNFα (**D**) in mouse brain homogenates in the presence and absence of HAEE measured by ELISA, *n* = 8. (**E**) HAEE’s (20 μM) effect on Aβ (100 nM) passage through a monolayer of bEnd.3 cells from the abluminal transwell compartment to the luminal compartment measured by ELISA at 24 h, *n* = 5. Effect of blocking antibodies to LRP1 and Pgp on Aβ levels in bEnd.3 cells in the absence (**F**) and presence (**G**) of HAEE measured by ELISA after 2 h of incubation with Aβ and HAEE, *n* = 3–4. Time course of Aβ levels in mixed primary culture cells (**H**), primary astrocyte cells (**I**), and HMC3 microglial cells (**J**) in the presence and absence of HAEE was measured by ELISA, *n* = 4 for H and I, *n* = 6 for J. HAEE’s effect on Aβ clearance rate in mixed primary culture cells (**K**) in primary astrocyte cells (**L**), and in HMC3 microglial cells (**M**) in the 24–48 h period. The clearance rate was calculated from the data in H, I, and J as the difference in Aβ level in the cells between the 48- and 24-hour time points divided by 24 h. Data are presented as mean of *n* independent experiments ± SD; *p* ≤ 0.05 (*), *p* ≤ 0.01 (**), *p* ≤ 0.001 (***), *p* ≤ 0.0001 (****).

We assessed the levels of Aβ and the pro-inflammatory cytokines IL-6 and TNFa in mouse brain tissue three days after the i.c.v. injection of oligomeric Aβ and a course of HAEE peptide injections (Figure 1A). Aβ level in the brain of mice treated with HAEE was reliably reduced compared to animals that did not receive the tetrapeptide (Figure 1B). The reduction in Aβ levels was associated with a 40% decrease in IL-6 levels induced by HAEE in mouse brain tissue (Figure 1C). However, HAEE had no effect on TNFα levels (Figure 1D).

Between 80–85% of Aβ is removed by transvascular BBB transport from the mouse brain [14]. To assess the effect of HAEE on Aβ clearance via the BBB, we measured Aβ passage through a monolayer of bEnd.3 endothelial cells in a transwell model *in vitro*. 24 h after adding Aβ to the abluminal compartment (“brain”), the rate of its passage across the BBB into the luminal compartment (“blood”) was significantly increased in the presence of HAEE (Figure 1E).

Aβ clearance from the brain across the BBB is mediated by the transporter proteins: Low-Density Lipoprotein Receptor Related Protein 1 (LRP1) and P-glycoprotein (Pgp) [14]. Using specific inhibitors, we showed that blocking LRP1 reduced Aβ uptake by endothelial cells by 30%, both the presence and absence of HAEE (Figure 1F,G). In contrast, inhibiting Pgp reduced intracellular Aβ levels by 70% with the addition of HAEE, compared to only 25% without HAEE (Figure 1F,G). Thus, both LRP1 and Pgp are involved in Aβ transport across the BBB; however, Pgp had a greater effect on amyloid uptake by endothelial cells in the presence of HAEE.

### 2.2. HAEE Enhances Aβ Clearance by Microglia and Reduces Aβ-Induced Microglial Activation

Clearance of molecules from the brain occurs not only through their transport into the bloodstream, but also by brain cells themselves [15]. We used a mixed primary culture of mouse brain cells to assess the effect of HAEE on the uptake and clearance of Aβ by the cells. The highest level of Aβ in the brain cells was observed at 4–24 h after addition of the peptide, and HAEE did not significantly affect the intracellular Aβ level during this period (Figure 1H). However, HAEE notably accelerated Aβ clearance by the cells at 24–48 h (Figure 1H,K).

Among the brain cells, astrocytes and microglia mostly uptake and dispose of Aβ and its oligomers [16,17]. HAEE decreased the level of intracellular Aβ in primary astrocyte culture after 24–48 h of incubation, compared to the cells treated with Aβ alone, but did not affect the rate of Aβ clearance (Figure 1I,L). In HMC3 microglial cells, HAEE significantly reduced the amount of intracellular Aβ after 24 h and 48 h of incubation compared to the cells not treated with HAEE (Figure 1J). In addition, HAEE doubled the rate of Aβ clearance in HMC3 cells (Figure 1M).

**Figure 2:**
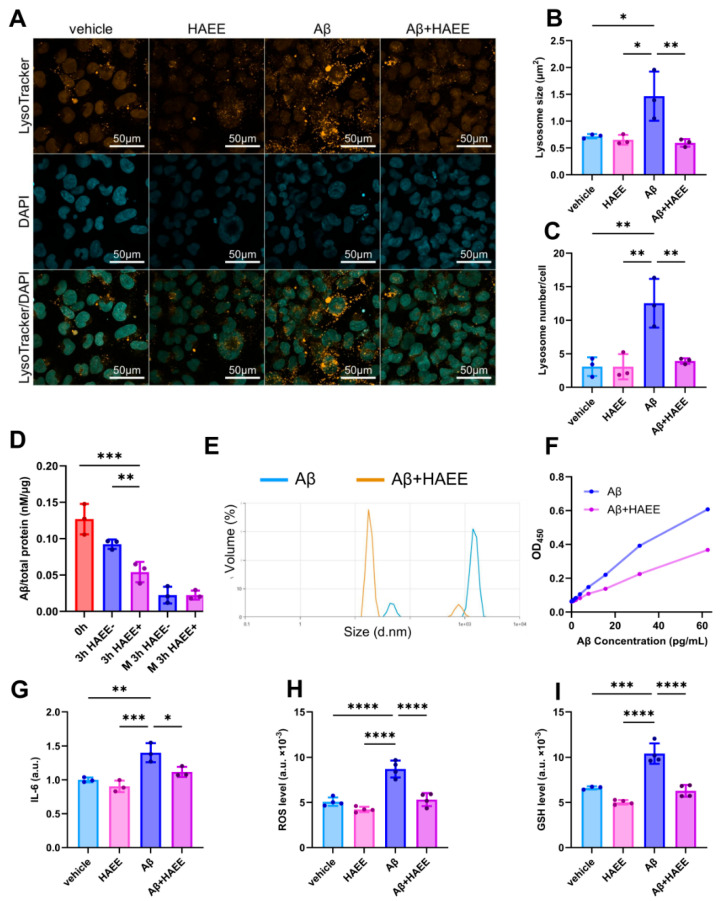
HAEE’s effect on Aβ degradation and related activation of HMC3 cells. (**A**) After 24-hour incubation with Aβ (1 μM) and HAEE (30 μM), lysosomes were visualized by LysoTracker Red DND-99 staining of HMC3 cells. NucBlue Live Cell Stain was used for nuclear staining. Scale bars, 50 μm. Size (**B**) and the number of lysosomes per cell (**C**) were calculated as the average of 10 cells per field of view, *n* = 3. (**D**) Effect of the exocytosis inhibition on HAEE-dependent Aβ degradation. Initially, HMC3 cells were treated with 1 µM of Aβ for 1 h and intracellular Aβ level was detected by ELISA (0 h). Then, the cells were washed, and HAEE and/or GW4869 were added to the cells for an additional 3 h (3 h HAEE+ or 3 h HAEE-). Additionally, Aβ was measured in the medium to control the inhibition of exocytosis (M 3 h HAEE+ or M 3 h HAEE-), *n* = 3. (**E**) Size of Aβ oligomers in the presence and absence of HAEE measured by DLS. Aβ (50 μM) was incubated for 24 h at 37 °C for oligomerization. Then, oligomeric Aβ was incubated with HAEE for an additional 24 h to evaluate the ability of HAEE to dissolve Aβ oligomers. (**F**) Level of Aβ oligomers in the presence and absence of HAEE measured by ELISA specific to oligomeric forms of Aβ. (**G**) HAEE’s effect on Aβ-induced HMC3 activation was assessed by the IL-6 level in the HMC3 supernatant using ELISA, *n* = 3. Intracellular ROS (**H**) and GSH (**I**) levels in HMC3 cells in the presence and absence of Aβ and HAEE were measured by flow cytometry, *n* = 4. Data are presented as mean of *n* independent experiments ± SD; *p* ≤ 0.05 (*), *p* ≤ 0.01 (**), *p* ≤ 0.001 (***), *p* ≤ 0.0001 (****).

Internalized Aβ is delivered to the lysosomes for further degradation [15]. To visualize this process, we performed lysosomal staining of HMC3 cells with LysoTracker Red DND-99 (Figure 2A). Microglial phagocytosis of oligomeric Aβ was accompanied by a significant reorganization of the lysosomal compartment: the average size of lysosomes increased by two times (Figure 2A,B) and their number by three times after Aβ uptake (Figure 2A,C) compared to control cells. However, 24 h HAEE treatment completely abolished the Aβ-induced changes in lysosomes and returned the lysosomal parameters to control levels (Figure 2A–C).

Decrease in the level of Aβ lysosomes in the presence of HAEE could be due to either faster Aβ degradation or more active exocytosis. To rule out the contribution of exocytosis to the HAEE-mediated reduction in Aβ levels, we used GW4869, an inhibitor of exosome biogenesis and release. Treatment with GW4869, which blocks Aβ exocytosis, did not prevent the effect of HAEE on the Aβ clearance in microglial cells. Intracellular Aβ level was reduced by 40% in HMC3 cells, treated with both HAEE and GW4869, compared to the cells treated with the inhibitor alone (Figure 2D).

Aβ degradation by microglial cells depends on its aggregation state [15,18]. Consequently, HAEE’s effect on microglial Aβ clearance could be linked to a change in the Aβ oligomeric state. Using dynamic light scattering (DLS), we showed that the addition of HAEE to a solution of Aβ oligomers reduced their average size (Figure 2E). Furthermore, using ELISA specific to Aβ oligomeric forms, we found that HAEE reduced the amount of Aβ oligomers (Figure 2F). Thus, HAEE not only prevented the formation of new Aβ aggregates [19], but also dissolved pre-formed oligomers.

Microglial cells significantly contribute to the development of neuroinflammation in the brain [20]. Accordingly, downregulation of the level of pro-inflammatory cytokine IL-6 in the brains of HAEE-treated mice (Figure 1C) may be associated with reduced microglial activation. Aβ-induced activation of HMC3 cells was accompanied by a significant increase in IL-6 level, while HAEE effectively inhibited this effect (Figure 2G, 24 h after Aβ treatment). Microglial activation is linked to oxidative stress [21]. Treatment of microglial cells with Aβ for 24 h caused a significant increase in the levels of reactive oxygen species (ROS) (Figure 2H) and reduced glutathione (GSH) (Figure 2I). At the same time, the addition of HAEE completely prevented the changes in these redox parameters (Figure 2G–I).

## 3. Discussion

The development of effective AD therapy is currently an active area of research. Modern therapeutic strategies target toxic forms of Aβ, which significantly contribute to the pathogenesis and severity of AD [22]. AD is characterized by the oligomerization and fibrillization of Aβ molecules [23]. Among all aggregate forms, soluble Aβ oligomers are considered the most toxic–they can spread throughout the brain, block cell receptors, exhibit synaptotoxicity, and their levels correlate with cognitive impairment [24]. Insoluble Aβ aggregates and fibrils damage cell membranes and form the core of amyloid plaques, the primary morphological hallmark of AD [25,26]. In addition to mechanically damaging cells, plaques trigger the activation of microglia and astrocytes, leading to the development of chronic, low-grade neuroinflammation [27]. Consequently, blocking the formation of Aβ oligomers/aggregates or stimulating their clearance may be an effective strategy against a range of AD symptoms, beyond merely reducing the Aβ level in the brain.

We previously developed the HAEE tetrapeptide, which crossed the blood–brain barrier (BBB), interacted with Aβ, and inhibited the formation of its zinc-dependent toxic aggregates [11,12,13]. In the present study, we established that HAEE also dissolved pre-formed Aβ oligomers (Figure 2E,F). Therefore, both the inhibition of aggregation and the removal of pre-existing Aβ aggregates contributed to the efficacy of HAEE in reducing the Aβ load in transgenic AD models *in vivo* [19,28].

Aβ aggregation makes it resistant to proteolytic degradation and disrupts its clearance from the brain [29]. Indeed, the HAEE-induced reduction in the size and number of Aβ aggregates contributed to the decreased level of Aβ in the brain (Figure 1B). HAEE enhanced the clearance of Aβ from the brain via several complementary pathways. We showed that HAEE increased Aβ passage from the brain to the blood across endothelial cells of the BBB (Figure 1E). Aβ transport across the BBB involves low-density lipoprotein receptors, including LRP1, or ABC transporters, in particular Pgp [30]. LRP1 interacted with HAEE [13] and participated in HAEE-dependent Aβ transport in b.End3 endothelial cells (Figure 1G). Shifting LRP1 transport towards transcytosis, increasing its levels, and enhancing LRP1-dependent Aβ efflux from the brain significantly reduces amyloid load and restores cognitive function in animals, with the therapeutic effect persisting for an extended period [31]. However, Pgp made a greater contribution to the HAEE-mediated transport of Aβ (Figure 1G). The activation of Pgp-dependent Aβ transport effectively reduces Aβ levels in the brain, while a decrease in Pgp activity may be considered an AD risk factor [32,33,34].

Clearance of Aβ occurs not only through its transport into the bloodstream or cerebrospinal fluid but also involves brain cells [16]. HAEE enhanced Aβ clearance in a primary brain cell culture (Figure 1H). Over 50% of brain cells are glial cells, specifically microglia and astrocytes [30]. Glial cells are involved in phagocytosis and degradation of Aβ, regulate neuroinflammation, and impairment of their normal function significantly contributes to AD pathogenesis [35]. We demonstrated that HAEE increased Aβ clearance by microglial cells but not by astrocytes (Figure 1I,J,L,M and Figure 2A–D). Enhancing Aβ phagocytosis by microglial cells represents an effective method for removing pathogenic Aβ species from the brain and is used in modern AD therapy [36,37]. Based on our findings, HAEE could both directly dissolve pre-existing Aβ aggregates (Figure 2E,F) and stimulate their degradation by microglial cells (Figure 1J,M and Figure 2D).

HAEE-related enhancement of Aβ clearance was accompanied by a reduction in the level of the pro-inflammatory cytokine IL-6 in the brains of the animals (Figure 1C). IL-6 activates the cGAS-STING pathway, which stimulates the pro-inflammatory activation of microglia and astrocytes and further contributes to neuroinflammation [38]. Moreover, lowering IL-6 levels restores cognitive function and decreases amyloid load in an AD animal model [38]. Elevated IL-6 levels are also associated with cognitive impairment in AD patients [39]. Thus, the HAEE-induced reduction in IL-6 may not only inhibit neuroinflammation, but also contribute to enhanced Aβ clearance and improvement in cognitive function in neuropathologies.

Chronic neuroinflammation in AD is largely mediated by activated microglia [2,5]. In response to Aβ deposits, microglia produce pro-inflammatory cytokines and reactive oxygen species, which exacerbate neuronal damage and create a vicious cycle that promotes disease progression [20]. HAEE inhibited both the Aβ-induced secretion of IL-6 by microglial cells and the alterations in its redox parameters that contributed to oxidative stress (Figure 2G–I). The activation of microglial cells correlates with cognitive impairment [40]. Therefore, HAEE may contribute to its improvement, a premise that requires verification in future studies.

In conclusion, the therapeutic effect of HAEE against Aβ-related neuropathologies is realized through several simultaneous pathways. HAEE not only inhibits the formation of toxic Aβ oligomers [28], but also promotes their disaggregation; increases its clearance across the BBB, mediated by LRP1 and Pgp; enhances Aβ degradation by microglia; and effectively decreases Aβ-induced neuroinflammation. This multifunctional effect of HAEE underscores its significant potential as an inhibitor of Aβ-related neuropathology.

ApoE4 is the primary genetic risk factor for the sporadic form of AD. Unlike other ApoE isoforms, ApoE4 impairs TREM2-dependent microglial phagocytosis, promotes microglial activation, and contributes to chronic neuroinflammation in AD [41,42]. Since HAEE was non-toxic [13], enhanced Aβ clearance by microglia, and reduced its activation and associated neuroinflammation (Figure 1J,M and Figure 2G–I), it may also be effective as a preventive AD therapy in ApoE4 carriers.

## 4. Materials and Methods

### 4.1. Preparation of Oligomeric Aβ and HAEE for Intracerebroventricular Injections

Oligomeric Aβ_1–42_ DAEFRHDSGYEVHHQKLVFFAEDVGSNKGAIIGLMVGGVVIA (Aβ, Peptide Specialty Laboratories GmbH, Heidelberg, Germany) was prepared as previously described [43]. Briefly, Aβ was dissolved in 0.1% NH_4_OH at a concentration of 1 mg/mL and sonicated for 1 min. The solution was then allowed to fibrilize for at least 1 h at 36 °C prior to injection.

The synthetic peptide Acetyl-HAEE-Amide (HAEE; chromatographic purity > 98%) was purchased from Synthon-Lab (St. Petersburg, Russia). The lyophilized peptide was dissolved in sterile artificial cerebrospinal fluid (ACSF; 127 mM NaCl, 1.0 mM KCl, 1.2 mM KH_2_PO_4_, 26 mM NaHCO_3_, 10 mM D-glucose, 2.4 mM CaCl_2_, 1.3 mM MgCl_2_, pH 7.4) to prepare a 10 mM stock solution, which was further diluted to the working concentration on the day of the experiment.

### 4.2. Animals

Experiments were performed on mature (8-month-old) female and male C57BL/6 mice. The animals were housed in the SPF vivarium of Belgorod State National Research University under standard conditions, including a standard diet, *ad libitum* access to food and water, a 12-hour light/dark cycle, an ambient temperature of +22 to +24 °C, and a relative humidity of 50–65%.

### 4.3. Surgical Procedure and Drug Administration

Mice were anesthetized with isoflurane (Laboratorios Karizoo, Barcelona, Spain) and placed in a stereotaxic apparatus. Body temperature was maintained with a heating pad. A guide cannula (22 G, RWD) was implanted over the left lateral ventricle (coordinates from bregma: AP = −0.7 mm; L = +1.4 mm; DV = −2.2 mm) according to the mouse brain atlas [44]. The cannula was fixed to the skull with dental acrylic.

After a recovery period, mice were randomly divided into two experimental groups using online GraphPad random number generator (Figure 1A, *n* = 8 per group): 1. The Aβ group received oligomeric Aβ and vehicle solution (ACSF). 2. The Aβ + HAEE group received oligomeric Aβ and the synthetic tetrapeptide HAEE (100 µM). The group allocation was unknown to the researchers who performed the neurosurgical procedures and ELISA.

On day 1, all mice received an intracerebroventricular (i.c.v.) injection of oligomeric Aβ (1 μL). Immediately after, mice received the first i.c.v. injection of either HAEE (1 μL) or ACSF (1 μL). Injections were repeated on days 2 and 3 (HAEE or ACSF only). All injections were performed on awake, freely moving mice through the guide cannula at a rate of 1 μL/min.

### 4.4. Sample Collection and Biochemical Analysis

Thirty minutes after the final injection on day 3, mice were decapitated, and whole brains were rapidly extracted and flash-frozen in liquid nitrogen. Frozen brain samples were homogenized in an ice-cold IP lysis buffer (ServiceBio, Wuhan, China) containing protease inhibitors (Thermo Fisher Scientific, Waltham, MA, USA). Homogenates were lysed for 30 min at 4 °C on a rotator and subsequently centrifuged at 12,000× *g* for 10 min at 4 °C. The resulting supernatants (brain tissue lysates) were collected for ELISA analysis.

### 4.5. Cell Cultures

A mixed primary culture containing neurons and glia was prepared from the cerebral hemispheres of postnatal day 2 (P2) C57BL/6 mouse pups according to a protocol [45]. Primary astrocyte culture was isolated from the mixed primary culture as described in [45]. Human microglial clone 3 (HMC3) cell line and mouse brain endothelial cell line (bEnd.3) were purchased from American Type Culture Collection (ATCC) and were cultured according to the handling information provided by ATCC.

### 4.6. Analysis of HAEE’s and Aβ’s Effects on the Cells

To evaluate HAEE’s and Aβ’s effects on the cells, a HAEE stock solution was prepared in PBS and synthetic Aβ was monomerized as described in [46]. Mixed primary culture, primary astrocyte culture, and HMC3 cells were treated with Aβ (1 µM) and/or HAEE (30 µM), which were dissolved in the culture medium without FBS. After incubation periods of 4, 24, and 48 h, the culture medium was aspirated, and cells were thoroughly washed with PBS to remove any non-specifically bound Aβ. Subsequently, cells were frozen in liquid nitrogen.

Frozen cells were lysed in IP lysis buffer (ServiceBio, Wuhan, China) containing protease inhibitors (Thermo Fisher Scientific, Waltham, MA, USA). The lysates were centrifuged at 12,000× *g* for 10 min at 4 °C, and the resulting supernatants were collected.

### 4.7. ELISA

Aβ levels in the brain and cell lysates were measured by sandwich ELISA using BAM113cc capture antibodies (50 ng/well, HyTest, Moscow, Russia) and peroxidase-labeled BAM7cc antibodies (100 ng/well, HyTest, Moscow, Russia) as described in [46]. Aβ level was normalized to the protein concentration in the samples, measured using a Pierce™ BCA Protein Assay Kit (Thermo Fisher Scientific, Waltham, MA, USA). The rate of Aβ degradation was calculated as the difference in normalized Aβ level in the cells between the 48-hour and 24-hour time points divided by 24 h. HMC3 supernatant was collected after 24 h treatment with Aβ and/or HAEE for interleukin-6 (IL-6) and tumor necrosis factor (TNFα) level’s measurement. IL-6 and TNFα in all samples were assessed using commercial ELISA kits (IL-6 Mouse Uncoated ELISA Kit, Mouse TNF alpha Uncoated ELISA Kit, Thermo Fisher Scientific, Waltham, MA, USA) according to the manufacturer’s instructions.

### 4.8. Oligomer-Specific ELISA

To measure Aβ oligomers using ELISA, two antibodies (a capture antibody and a detector antibody) targeting the same epitope are used. This method therefore allows for the detection of predominantly oligomeric forms of Aβ, which have epitopes available for binding to both the detection antibody and the capture antibody. This type of ELISA has been shown to be capable of detecting Aβ oligomers ranging in size from dimers to much larger aggregates, while Aβ monomers are not detected [47].

BAM7cc antibodies (HyTest, Moscow, Russia), which recognize the N-terminus of Aβ, were added to a 96-well ELISA plate (Wuxi NEST Biotechnology, Wuxi, China) in a volume of 100 μL (0.5 ng/μL) and incubated overnight at +4 °C. The wells of the plate were washed 3 times with 300 μL of PBST (0.05% Tween20) and blocked in 1% BSA (Diaem, Moscow, Russia) in PBST at room temperature and shaken for 1 h. The plate was then washed three times with 300 μL of PBST, and samples were added at a volume of 100 μL per well. The plates were incubated for 2 h at room temperature with shaking, washed 3 times with 300 μL of PBST, and HRP-conjugated BAM7cc antibodies (HyTest, Moscow, Russia) were added at a volume of 100 μL per well (1 ng/μL). The plates were incubated for 1 hour at room temperature with shaking, washed 5 times with 300 μL of PBST, and analyzed using TMB (Diaem, Moscow, Russia). Absorbance was measured at 450 nm using a Multiskan FC Microplate Photometer (Thermo Fisher Scientific, Waltham, MA, USA).

### 4.9. BBB Transwell Model

Transport of Aβ across the in vitro model of the BBB was measured as described in [40]. For the experiments, mouse bEnd.3 cells were seeded onto the upper surface of transwell inserts (Greiner Bio-One, Kremsmünster, Austria; pore size 0.4 μm) at a density of 70,000 cells per insert and cultured for 7 days to form a confluent monolayer. The volume of medium in the luminal (upper) compartment was 750 μL and in the abluminal (lower) compartment was 1 mL. Prior to the addition of HAEE and Aβ, both compartments were washed with serum-free DMEM to remove residual FBS.

The experiment was designed to model the clearance of Aβ from the brain (abluminal-to-luminal transport) (Figure 1B). Accordingly, the abluminal (lower) compartment was filled with 750 μL of serum-free DMEM containing 100 nM of Aβ or 100 nM of Aβ with 20 μM HAEE. The luminal (upper) compartment contained 700 μL of serum-free DMEM. The plates were incubated at 37 °C and 5% CO_2_. Samples were collected from the luminal compartment at 24 h. The Aβ transport rate was calculated as the ratio of the amount (pmol) of Aβ in the upper compartment measured by sandwich ELISA to the incubation time (min) and transwell area (cm^2^). To control model BBB integrity, paracellular permeability of the bEnd.3 monolayer was assessed using the fluorescent label sodium fluorescein (Sigma-Aldrich, St. Louis, MO, USA) in each experiment as described in [46].

### 4.10. Inhibitory Assay

To study the mechanism of Aβ transport with HAEE, bEnd.3 endothelial cells were cultured in a 24-well plate (Greiner Bio-One, Kremsmünster, Austria) until confluent. Cells were incubated with blocking antibodies to LRP or Pgp receptors (10 μg/mL, Santa Cruz Biotechnology, Dallas, TX, USA) for 1 h at +37 °C. Then, the wells of the plate were filled with 300 μL of a solution containing 100 nM Aβ or 100 nM Aβ with 20 μM HAEE. After 2 h, the cells were washed 3 times with PBS, frozen in liquid nitrogen, and lysed in IP lysis buffer (ServiceBio, Wuhan, China) with the addition of a protease inhibitor (Thermo Fisher Scientific, Waltham, MA, USA) for 1 h at +4 °C with stirring. The cell lysate was centrifuged at 16,000× *g*, +4 °C, 10 min and the supernatant was collected. The amount of total protein in the lysates was determined using a BCA assay kit (Sigma-Aldrich, St. Louis, MO, USA) according to the manufacturer’s protocol. The concentration of Aβ in the samples was measured by ELISA using the method described above.

### 4.11. Aβ Degradation Assay

HMC3 cells were treated with 1 µM Aβ in serum-free culture medium for 1 h to allow for peptide internalization. Cells were then washed to remove extracellular Aβ. To analyze Aβ degradation, the cells were further incubated for 3 h in a medium containing 10 µM GW4869 (an inhibitor of exosome biogenesis and release), either with or without 30 µM HAEE. Intracellular Aβ level was measured by ELISA after the 1-hour uptake period and again after the 3-hour degradation phase. Aβ released into the cell culture medium during the degradation phase was also quantified by ELISA.

### 4.12. Flow Cytometry Analysis

Reactive oxygen species (ROS) and reduced glutathione (GSH) levels were measured after 24 h incubation of Aβ with HMC3 cells. The cells were stained with dihydrorhodamine 123 (Ex/Em = 507/525 nm) or monobromobimane (Ex/Em = 395/495 nm) according to ThermoFisher Scientific’s instructions. One minute prior to analysis, 10 μg/mL of propidium iodide (Ex/Em = 535/617 nm, Sigma, St. Louis, MO, USA) was added to the cell samples to evaluate the number of dead cells in the population. The analysis was performed using a BD LSR Fortessa flow cytometer (Becton Dickinson, Franklin Lakes, NJ, USA) and FlowJo^TM^ Software v.10.8.1 (Becton Dickinson, Franklin Lakes, NJ, USA).

### 4.13. Lysosomal Staining and Visualization

HMC3 cells were treated with 1 µM Aβ, 30 µM HAEE, or their combination for 24 h. Two hours prior to the completion of the incubation period, LysoTracker Red DND-99 (Thermo Fisher Scientific, Waltham, MA, USA) was added to the culture medium at a final concentration of 50 nM to label acidic compartments, including active lysosomes. Immediately before live-cell imaging, cell nuclei were counterstained with NucBlue Live Cell Stain (Thermo Fisher Scientific, Waltham, MA, USA) according to the manufacturer’s instructions. Fluorescent images were acquired using a Nikon Eclipse Ni-U (Nikon Corp., Tokyo, Japan) microscope with appropriate filter sets.

### 4.14. Dynamic Light Scattering (DLS)

The size distribution of Aβ oligomers was characterized by DLS using a Zetasizer Nano ZS instrument (Malvern Panalytical, Malvern, UK). Oligomeric Aβ was prepared as described in Section 4.6 and further incubated at a concentration of 50 µM in PBS for 24 h at 37 °C to promote oligomerization. To assess the effect of HAEE, the peptide was added to the pre-formed Aβ oligomers at a final concentration of 500 µM (dissolved in PBS), and the mixture was incubated for an additional 24 h at 37 °C. A control sample containing Aβ without HAEE was processed in parallel under identical conditions. Immediately prior to analysis, samples were briefly centrifuged to sediment any large aggregates and transferred to a disposable polystyrene cuvette (Sarstedt, Nümbrecht, Germany). Measurements were performed at 25 °C, and the hydrodynamic diameter (reported as Z-average) and polydispersity index (PDI) were derived from the correlation function using the Zetasizer Software version 7.13 (Malvern Panalytical, Malvern, UK). Each sample was measured in triplicate.

### 4.15. Statistical Analysis

The mean values ± standard deviation (SD) were calculated for each dataset and are presented in the figures. Statistical differences between the experimental groups were analyzed using two-tailed Student’s *t*-test or two-way ANOVA with Tukey’s, * *p* < 0.05, ** *p* < 0.01, *** *p* < 0.001, **** *p* < 0.0001. Statistical analysis was performed by the GraphPad Prism v10.6.0.

## Data Availability

All relevant data are contained within the manuscript.

## Abbreviations

AD—Alzheimer’s disease; ATCC—American Type Culture Collection; Aβ—beta-amyloid; BBB—blood–brain barrier; bEnd.3—Mouse brain endothelial cell line; DLS—dynamic light scattering; DMEM—Dulbecco’s modified eagle medium; ELISA—Enzyme-linked Immunosorbent Assay; GSH—reduced glutathione; HAEE—Ac-HAEE-NH_2_; HMC3—Human Microglial Clone 3; i.c.v.—intracerebroventricular; IL-6—Interleukin-6; LRP1—lipoprotein receptor-related protein 1; MEM—Minimum Essential Medium; Pgp—P-glycoprotein; ROS—reactive oxygen species; TNFα—Tumor necrosis factor.
